# Dissociating dynamic probability and predictability in observed actions—an fMRI study

**DOI:** 10.3389/fnhum.2014.00273

**Published:** 2014-05-07

**Authors:** Christiane Ahlheim, Waltraud Stadler, Ricarda I. Schubotz

**Affiliations:** ^1^Department of Psychology, Institute of Psychology, University of MünsterMünster, Germany; ^2^Motor Cognition Group, Max Planck Institute for Neurological ResearchCologne, Germany; ^3^Department of Sport and Health Science, Technische Universität MünchenMunich, Germany; ^4^Department of Cognitive Neurology, Max Planck Institute for Human Cognitive and Brain SciencesLeipzig, Germany

**Keywords:** statistical learning, action observation, orbitofrontal cortex, dmPFC, fMRI, information theory

## Abstract

The present fMRI study investigated whether human observers spontaneously exploit the statistical structure underlying continuous action sequences. In particular, we tested whether two different statistical properties can be distinguished with regard to their neural correlates: an action step's predictability and its probability. To assess these properties we used measures from information theory. Predictability of action steps was operationalized by its inverse, conditional entropy, which combines the number of possible action steps with their respective probabilities. Probability of action steps was assessed using conditional surprisal, which increases with decreasing probability. Participants were trained in an action observation paradigm with video clips showing sequences of 9–33 s length with varying numbers of action steps that were statistically structured according to a Markov chain. Behavioral tests revealed that participants implicitly learned this statistical structure, showing that humans are sensitive toward these probabilistic regularities. Surprisal (lower probability) enhanced the BOLD signal in the anterior intraparietal sulcus. In contrast, high conditional entropy, i.e., low predictability, was correlated with higher activity in dorsomedial prefrontal cortex, orbitofrontal gyrus, and posterior intraparietal sulcus. Furthermore, we found a correlation between the anterior hippocampus' response to conditional entropy with the extent of learning, such that the more participants had learnt the structure, the greater the magnitude of hippocampus activation in response to conditional entropy. Findings show that two aspects of predictions can be dissociated: an action's predictability is reflected in a top-down modulation of attentional focus, evident in increased fronto-parietal activation. In contrast, an action's probability depends on the identity of the stimulus itself, resulting in bottom-up driven processing costs in the parietal cortex.

## Introduction

When we observe another person's action, we are quite accurate at predicting what is going to happen next (Stadler et al., 2011; Zacks et al., 2011). But how do we know? Theoretically, we can be taught that an action A is typically followed by action B, as for instance when we learn how to bake a cake. However, we can also acquire knowledge about the structure of action sequences through statistical learning (Avrahami and Kareev, 1994; Baldwin et al., 2008). Statistical learning describes a mechanism of learning about associations between events through repeated experience of their co-occurrence or succession either in time or space (Turk-Browne et al., 2009; Fiser et al., 2010). Thereby, we learn about two statistical measures of actions that we can exploit to predict upcoming steps, given the current action step we observe: the number of possible action steps and their probabilities. The number and probability of the alternatively possible action steps at a particular moment (i.e., the degree of weighted branching at a node in the action sequence) determines the action's predictability, while in contrast to that, an action step's probability depends on the particular action step alone. For example, taking a banana is most often directly followed by peeling it, while taking an apple can be directly followed by a larger number of action steps, as, e.g., eating the apple, peeling, or cutting it. So after seeing someone grasping a banana, predictability of the next step is high, as only one action step is highly probable, while predictability of the next action step is lower after seeing someone taking an apple. To keep with the above example, despite the higher predictability after seeing someone grasping a banana, the probability of putting the banana in a lunchbox could be the same as putting an apple in a lunchbox. From a neuroscientific perspective, a differentiation between the two aspects is crucial: while an event's probability reflects how (un-)expected its occurrence was and hence, how much an observer needs to adapt his previously built expectations, predictability influences how precise an observer's expectations could be.

As this example illustrates, predictability and probability both quantify the statistical structure of actions, or more generally, events. While predictability (or its inverse, entropy, cf. Shannon, 1948) derives from the number of possible events and their respective probabilities, probability of an event (or its inverse, surprisal, cf. Tribus, 1961) refers to the event alone. Thus, predictability of an event can vary independently of its absolute probability. In actions, predictability is lowest at action boundaries (Zacks et al., 2011), depending on the weighted degree of branching at the node in the action sequence. The independence of predictability and probability is reflected by the observation that they differently affect encoding of stimulus streams (Strange et al., 2005; Harrison et al., 2006; Bornstein and Daw, 2012).

Various research has provided evidence that people are able to implicitly learn the statistical structure underlying incoming stimulus streams in both visual as well as auditory material (e.g., Hunt and Aslin, 2001; Saffran, 2001; Harrison et al., 2006; Swallow and Zacks, 2008; Bornstein and Daw, 2012; Paraskevopoulos et al., 2012; see Perruchet and Pacton, 2006 for a review). However, so far previous studies on statistical learning in actions have focused on learning of successions of separate action clips (Avrahami and Kareev, 1994; Baldwin et al., 2008; Swallow and Zacks, 2008), while evidence for statistical learning in dynamic action sequences is still lacking.

Building on this prior work, the goals of this study were two-fold. First, we aimed at establishing a role of statistical structure in the perception of continuous action sequences in general. Second, we wanted to address the question of neural correlates of predictability and probability of action steps at the current position of an action sequence. It has been shown that predictions of abstract visual events and actions rely in parts on identical brain sites, but engage also different ones (Schubotz and von Cramon, 2008). As most studies on predictability and probability in event streams made use of abstract visual stimuli, we aimed at extending knowledge on this further and examine, if the respective networks overlap or differ in their components.

**Figure 1:**
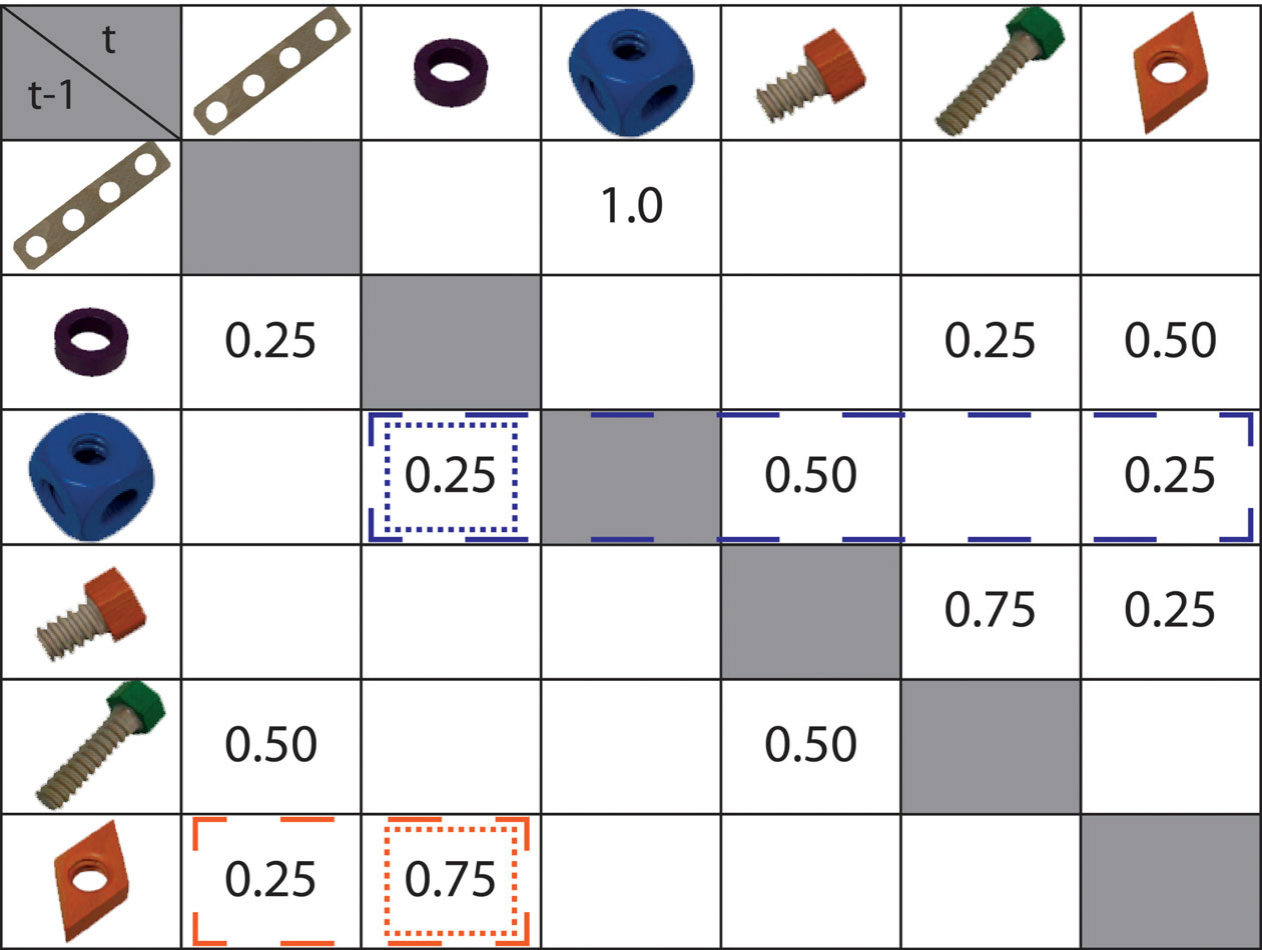
**Markov chain ruling the presented action sequences**. Rows depict the first objects of a transition (*t* - 1), e.g., the board (first row) was always (*p* = 1.0) followed by a cube (third column), whereas the cube (third row) could be followed by a washer (*p* = 0.25), a short screw (*p* = 0.50), or a screw nut (*p* = 0.25). Conditional surprisal of an action step depended on its probability given the preceding action step only. An example is highlighted in the figure: cells surrounded by dotted lines determine the surprisal assigned to the washer after a screw nut (orange) or a cube (blue). In contrast, an action step's conditional entropy depended on its own probability and the probability weights of alternative action steps. For instance, cells surrounded by dashed lines determine the conditional entropy assigned to the washer after the screw nut (orange) or cube (blue).

To be able to dissociate predictability and probability of actions, we created action sequences according to a first-order Markov structure. That is, the predictability and probability of one certain action step depended on the preceding action step, i.e., they were conditional on their predecessor. We implemented two distinct measures for each quantity. Effects of action probability were measured as conditional surprisal, whereas action predictability was operationalized as conditional entropy (Shannon, 1948; Cover and Thomas, 1991). Conditional entropy combines the number of possible alternative action steps and their respective probabilities (for further details, see Materials and Methods and Figure 1).

We expected to find effects of the conditional surprisal of an action step in a lateral network often engaged by observing actions, including the premotor cortex, parietal sites, and the posterior temporal cortex (Jeannerod, 2001; Schubotz and von Cramon, 2004, 2008; Caspers et al., 2010). This network, also referred to as action-observation network, shows an increased response during the encounter of unexpected actions (Schiffer et al., 2013) and is furthermore also correlated with the surprisal of an abstract event (Strange et al., 2005; Bubic et al., 2011). Hence, we hypothesized activation in the action-observation network to show a higher activation for action steps with a higher surprisal.

The degree of predictability of abstract stimuli has been found to draw on attentional and memory systems (Strange et al., 2005; Bornstein and Daw, 2012; Nastase et al., 2014), showing higher activations for less predictable stimuli. In line with this, Schubotz et al. (2012) found increased activation in left dorsolateral prefrontal cortex (dlPFC), parahippocampal gyrus, and posterior angular gyrus (AG) when observers noticed an action boundary in everyday actions (i.e., when predictability was low), and interpreted this as reflecting a shift of spatial attention that is guided by long-term action knowledge. However, this study did not address quantified predictability that results from the number and probability-balance of possible upcoming action steps. First evidence for a quantitative effect of the number of probable actions has been provided by Schiffer et al. (2012), who found an increase of activity in the hippocampal formation as the number of possible action steps increased and hence, predictability decreased. Based on these previous findings, we hypothesized activation in the hippocampal formation and the AG to correlate with predictability of observed action steps. Predictability was measured as conditional entropy, which is the inverse of predictability. Thus, we expected a positive correlation of the BOLD signal with conditional entropy. In psychological terms, conditional entropy can also be translated as conflict or uncertainty, as both rise, as more possible and probability-balanced alternatives are at hand (cf. Berlyne, 1957). Research on response conflict as well as on decisional uncertainty suggests a role of the posterior dorsomedial frontal cortex in adapting behavior to such situations (Ridderinkhof et al., 2004; Volz et al., 2005; Mushtaq et al., 2011). We thus hypothesized activation in the dorsomedial prefrontal cortex (dmPFC) to be positively correlated with the conditional entropy of upcoming actions.

## Materials and methods

### Participants

Seventeen healthy right-handed participants volunteered in the fMRI study [mean age 25 (20–34) years, eight female, 14 students]. They were recruited from the volunteer database of the Max-Planck-Institute for Human Cognitive and Brain Science. No participant reported a psychiatric or neurological disorder. They gave written informed consent and received a financial reimbursement of 10€ per hour. The local Ethics Committee of the University of Leipzig approved the experimental standards. Two volunteers had to be excluded, one due to technical difficulties and one due to poor performance in the control task (score below two standard deviations from mean) and self-reported periods of sleep (results did not change qualitatively if participant was included in analysis). All following analyses of functional and behavioral data are thus based on data from 15 participants [eight female, mean age 25 (20–34) years].

### Stimulus material

The stimulus material consisted of videos showing sequences of action steps using objects of the constructional toy Baufix® (Figure 1). Overall, six different objects were used: a board, a cube, a long screw, a short screw, a nut, and a washer. An action step was defined as the grasping and mounting of one object. Each object was always manipulated in the same way: the cube was screwed on the scaffold, the long screw was put through a hole of a board, washer and boards were placed on long screws, screw-nuts were attached to screws, and short screws were screwed into cubes. Action steps were performed in a naturalistic manner and hence differed in their length and speed of movement.

Videos showed sequences comprised of varying combinations and numbers of these six action steps. The transitions between action steps followed a Markov chain (see Figure 1) and were the same for all subjects. Transition probabilities between action steps were pre-defined and ranged from *p* = 0.25 to *p* = 1. Except mounting of the cube, which was always preceded by the same action step, each action step was preceded by one out of two to three different action steps and depending on the preceding action step, one, two or three different action steps were concurrently possible, causing different values of conditional entropy and surprisal (see section “Contrast Specification”). Importantly, this statistical structure enabled us to disentangle values of conditional entropy and surprisal from identity of action steps and involved objects, as well as the characteristics of the action steps as speed of movement and length of manipulation. Repetitions of action steps within a sequence were possible but were not correlated significantly with our measures of interest (correlation with conditional entropy *r* = 0.03, correlation with conditional surprisal *r* = −0.12). Direct repetitions of action steps did not occur. To implement the Markov chain, 74 construction sequences were compiled. Action sequences differed in the number of action steps they comprised, ranging from three to seven (*M* = 4.89, *SD* = 1.28), and their overall presentation duration (*M* = 20.12 s, *SD* = 6.04). Note that constructions did not aim to reach a specific, pre-defined overarching goal, as for instance building a vehicle.

Overall, each action step was presented about 60 times (58–63, *M* = 60.33), so that all action steps had a comparable base rate. We moreover balanced how often an action step emerged as the first or the last step of a construction sequence (first: 10–15, *M* = 12.5; last: 10–15, *M* = 12.33).

**Figure 2:**
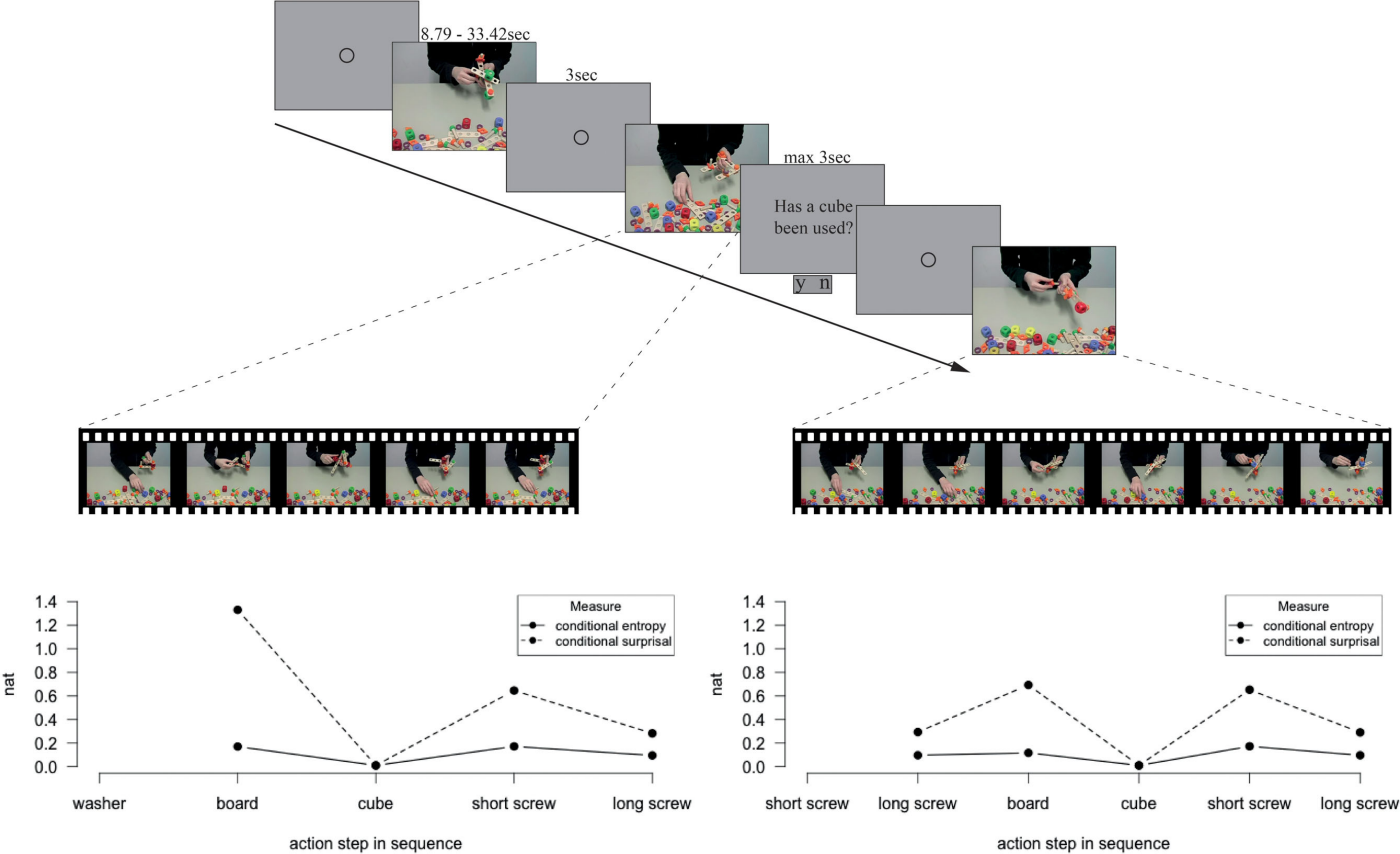
**Experimental course and exemplary distribution of conditional entropy and conditional surprisal during an action sequence**. A fixation circle announced each video and 48% of the videos were followed by a two alternative forced choice question. Feedback on correctness of responses was only given during the training sessions. Within each action sequence, values of conditional entropy and conditional surprisal were ascribed to the action steps, depending on the preceding action step.

To have ample degrees of freedom for the construction process, the first action step was always performed on a prepared “starting” scaffold consisting of various different mounted objects (as can be seen in Figure 2). Sequences started at the moment the actor lifted the scaffold and ended when the scaffold was placed on the table again. In sum, five different starting scaffolds were employed. Each of the 74 action sequences was filmed once with each of these five scaffolds (resulting in 370 videos altogether), so that participants never saw the exact same shot of one action sequence twice. Hence, expectations within action sequences could only be based on the employed transition probabilities between action steps.

Videos were filmed from the third person perspective with no zooms or camera motions. The focus was on the center of the table and offered a good view of the actor's hands, but not the head, and numerous different objects in the foreground (see Figure 2). The software iMovie '09 (Apple, Inc., Cupertino, CA) was used for video processing.

Randomization of order of the sequences during the experiment was constrained by allowing maximal two repetitions of the used scaffold, the sequence length as well as the first and last element of the sequence. Additionally, the cases of the former sequence being a subsequence of the latter and vice versa were excluded.

### Experimental procedures

The experiment took place on three successive days. The first two sessions served as training to provide participants with implicit knowledge of the underlying statistical structure of the action sequences. On the third day, participants first underwent the fMRI experiment. Afterwards, they took part in two post-tests, which tested their implicit knowledge of the action syntax. The experiment was programmed and run on Presentation 12.0 (Neurobehavioral Systems, San Francisco, CA, USA).

### Training sessions (day 1 and 2)

During each of the two 35-min training sessions, participants were exposed once to each of the 74 sequences. Participants were exposed to a different randomization of movies in each training session.

Participants were instructed to watch the videos carefully and to answer the occasional questions concerning the previous video. Questions appeared after 36 of the 74 video clips (48%). It is important to note that participants did not receive explicit learning instructions at any point of the training (or the subsequent fMRI session), nor were they told that there was a certain systematic concerning the statistical structure of the action sequences. No cover story was provided.

Before starting the training, participants were familiarized with the six different objects as well as with the possible questions (e.g., “Has a long screw been used?”). During training, participants had to press the right mouse button (i.e., middle finger of the right hand) corresponding to the answer “no” and the left mouse button (i.e., right index finger) corresponding to “yes.” Half of the questions required an affirmative answer.

The videos were displayed in front of a gray background in the middle of a computer screen (subtending approximately 12.5^*^10° of visual angle). A fixation circle announced videos for 3 s (or variable length after question trials; see Figure 2 for an illustration of the trial course). Questions were presented for 3 s or until the first response; after question trials, the duration of the fixation circle was adapted to compensate for different reaction times (with keeping a minimum duration of 2 s). Questions were followed by a feedback of 2 s indicating correct (“+”), incorrect (“−”), or delayed (“/”) responses.

### Functional MRI session (day 3)

The task in the fMRI session was identical to the training sessions, except that no feedback was provided. Participants were informed about this difference beforehand.

In addition to the experimental block, we ran four functional localizers adapted from Wurm and Schubotz (2012) after the main experiment so as to identify brain regions related to the processing of Baufix® objects, other tools, motion, and human body (see Supplemental Material for Analysis and Results).

Following the functional scanning, two post-tests assessed participants' implicit knowledge of the action syntax. During the first post-test, a paper–pencil test, six video clips were presented in randomized order. These clips ended after one object had been used and the actor reached for a second one. The participants' task was to mark those objects out of the possible six that they expected to be used next and to indicate their respective probability. To this end, they had to assign overall eight crosses among the six items. For instance, if participants saw a clip in which the long screw had been used and they expected the board and the short screw afterwards with equal probability, they assigned four crosses to each of them. The number of eight crosses was chosen to allow participants to select up to all six possible objects and to weight them accurately (each cross corresponded to *p* = 0.125). In the second post-test, participants were presented each possible succession of two of the six objects and were asked to enter a value between 0 and 100% representing how likely they considered each succession with regard to the previously seen videos. Responses were given via keyboard, and participants could revise their answer before finally submitting it.

After completing the post-tests, participants were interviewed to further assess if they have consciously noticed the statistical structure of the presented action sequences and if so, to which degree they were able to specify the structure. To this end, they were asked verbally if they have noticed any associations between the action steps and if so, if they could define them. Furthermore, they were asked if the actions were predictable for them.

### Behavioral data analysis

The statistical analysis of the two post-tests was performed with SPSS Statistics version 20.0 (SPSS Inc. Chicago, Illinois, USA). An α-level of 0.05 was defined as statistical threshold.

First, we aggregated separately for each post-test for each participant the estimated probabilities of the transitions, depending on the underlying level of implemented probabilities (0, 0.25, 0.50, 0.75, or 1.0), e.g., we calculated the average estimated probability for all transitions with the same true probability. Those aggregated probability estimates were entered in a separate repeated measures analysis of variance (RM-ANOVA) with the factor PROBABILITY (0, 0.25, 0.50, 0.75, 1.0) for each post-test. When the assumption of sphericity was violated, a Greenhouse-Geisser correction was used to adjust the degrees of freedom.

### Functional data analysis

#### Contrast specifications

Predictability of action steps was manipulated by the number of possible next action steps and their respective probabilities. Conditional entropy (H) provides a measure that takes both aspects into account and is higher, the lower the predictability is. In contrast, probability of the factually occurring action step was modeled as conditional surprise (I, surprisal hereafter, cf. Tribus, 1961), which is the negative logarithm of an action step's probability. The applied modeling of conditional entropy and conditional surprisal was in close proximity to the approach taken by previous studies (Strange et al., 2005; Harrison et al., 2006; Bornstein and Daw, 2012; Schiffer et al., 2012).

Conditional entropy and surprisal are only partially statistically independent, because the probability of a single action step decreases as the number of possible action steps increases. The advantage of modeling correlated parameters simultaneously in one general linear model (GLM) is that any variance that is explained by both parameters will not be erroneously assigned to exclusively one of them. At the same time, this approach has the disadvantage that it does not show areas that are truly modulated by both conditional entropy and surprisal. That is, commonalities will be underestimated (false negatives). To avoid this latter fallacy, we additionally tested for effects of conditional entropy and conditional surprisal by employing a separate design for each and provide results in the Supplementary Materials. Both approaches resulted in similar results, but showed also some differences.

#### Calculating probabilities: bayesian modeling approach

We modeled the neural responses according to an ideal observer model, which tracks the number of occurrences of events and calculates probabilities based on all occurrences (cf. Strange et al., 2005; Harrison et al., 2006; Bornstein and Daw, 2012; Schiffer et al., 2012). Hence, the probability *p* of a single item *x_t_* was calculated as the number of occurrences *n* of item *x_t_* divided by the sum of all items *x_i_* that have appeared so far (see Equation 1). The addition of the value 1 shapes a Dirichlet function.

$$p\left(x_{t}\right)=\frac{n\left(x_{t}\right)+1}{\sum_{i}x_{i}+1}$$

Equation 1. Calculation of Bayesian probabilities.

The ideal observer model included the training sessions, so transition probabilities were already taken as established at the beginning of the fMRI session. Since all action steps had a similar base rate, we did not calculate the surprisal of the occurrence of an action step *per se*, i.e., *p*(*x_t_*). Instead, we calculated the conditional surprisal ascribed to a transition, i.e., the occurrence of an action step, given that a particular action step had happened before, *p*(*x_t_*|*x*_*t* − 1_) (Equation 2). Values for surprisal ranged from 0.01 to 1.38 (*M* = 0.63, *SD* = 0.49).

$$I\left(x_{t}|x_{t\;-\;1}\right)=-log\;p\left(x_{t}|x_{t\;-\;1}\right)$$

Equation 2. Calculation of conditional surprisal.

In analogy, we did not calculate the entropy ascribed to the underlying Markov chain of the action sequences, but focused on the specific conditional entropy (Cover and Thomas, 1991). Conditional entropy refers to the entropy ascribed to an upcoming event when the prior event is taken into account. It describes the (on average) expected surprise. It is calculated as mean surprise of all possible events *x_t_* given that *x*_*t* − 1_ had occurred, standardized on the probability of the prior event *p*(*x*_*t* − 1_) (Equation 3). Values ranged from 0.01 to 0.72 (*M* = 0.11, *SD* = 0.05). Correlation of both parameters was *r* = 0.67.

$$H\left(x_{t}|x_{t\;-\;1}\right)=-p\left(x_{t\;-\;1}\right)\sum_{i}p\left(x_{t}^{i}|x_{t\;-\;1}\right)\;*\;log\;p\left(x_{t}^{i}|x_{t\;-\;1}\right)$$

Equation 3. Calculation of conditional entropy.

### fMRI data acquisition and analysis

A 3 T Siemens Magnetom Trio (Siemens, Erlangen, Germany) system equipped with a standard birdcage headcoil was used in the functional imaging session. Participants lay supine in the scanner with their right hand on a four-button response-box and their index and middle finger placed on the two appropriate response buttons. Response contingencies were the same as in the training sessions. Form-fitting cushions were used to prevent participants from head or arm movements and they were provided with earplugs to attenuate scanner noise. The experiment was presented via a mirror that was built into the headcoil and adjusted individually to provide a good view of the entire screen.

Prior to functional imaging, 28 slices of anatomical T1-weighted MDEFT images (4 mm thickness, 0.6 mm spacing) and a fieldmap scan, consisting of a gradient-echo readout with 24 echoes and an inter-echo time of 0.95 ms, were acquired. During functional imaging, 28 axial slices (126.8 mm field of view, 4 mm thickness, 0.6 mm spacing; in-plane resolution of 3 × 3 mm) parallel to the bi-commissural line (AC-PC) were collected using a single-shot gradient echo-planar (EPI) sequence (2000 ms repetition time; echo time 30 ms, flip angle 90°, serial recording), sensitive to BOLD contrast.

To improve the localization of activation foci, high-resolution 3D T1-weighted whole brain MDEFT sequences (175 sagittal slices, 1 mm thickness) were recorded for each participant in a separate session.

Functional data were processed using the LIPSIA software package, version 2.1 (Lohmann et al., 2001). First, a distortion correction using the field map scan was performed. To correct for temporal offsets between the slices acquired in one scan, a cubic-spline interpolation was used. Thereafter the data were motion-corrected with the 50^th^ time-step as reference and six degrees of freedom (three rotational, three translational). A highpass filter of 1/70 or 1/55 Hz (different between participants) was applied to remove low-frequency signal changes and baseline drifts. Highpass filter width was determined by an optimization algorithm implemented in the LIPSIA package.

Functional data slices were aligned with a 3D stereotactic coordinate system. To that end, in a first step the matching parameters (six degrees of freedom, three rotational, three translational) of the T1-weighted 2D-MDEFT data onto the individual 3D-MDEFT reference set were calculated. The thereby gained transformation matrix for a rigid spatial registration was normalized to a standardized Talairach brain size (*x* = 135, *y* = 175, *z* = 120 mm; Talairach and Tournoux, 1988) by linear scaling. Thereafter the normalized transformation matrices were applied to the functional slices, in order to transform them using trilinear interpolation and align them with the 3D-reference set in the stereotactic coordinate system. After the described processing, the spatial resolution of the functional data was 3 ^*^ 3 ^*^ 3 mm (27 mm^3^). A spatial Gaussian filter of 5.65 mm full width at half maximum (FWHM) and a standard deviation of 0.8 mm was applied to the data.

#### Design specifications

We modeled the parametric contrasts of conditional surprisal and conditional entropy time-locked to the beginning of a new action step. Onsets were defined as the starting of the hand movement to the next object. If two events were separated by less than 2 s (i.e., less than one TR), only the first one was included in the GLM, while the second was ignored and treated as part of the implicit baseline. To control for variance due to action observation in general, we also modeled the video clips as epochs (mixed design). The parametric contrasts of conditional entropy and conditional surprisal contained 219 events with a mean difference between events of 7.93 s (5.01 s *SD*), which were selected from 74 video epochs.

The statistical evaluation was based on the least-square estimation using the GLM for serially auto-correlated observations and a temporal Gaussian filter with a FWHM of 4 s was applied to deal with auto-correlation (Friston et al., 1995; Worsley and Friston, 1995).

To calculate the parametric effects of conditional surprisal and conditional entropy, the design matrix was generated with a delta function and its first derivative, convolved with the hemodynamic response function (gamma function) (Glover, 1999). The BOLD signal was analyzed time-locked to the specific events. The design matrix included six regressors: one for the main effect of action onsets with an amplitude of one, one for the parametric effect of conditional entropy, one for conditional surprisal, with an amplitude according to the respective measure, and two each with an amplitude of one for question trials and video epochs. The duration of action steps was included as a regressor of no interest. Besides the video epochs and the question trials, all events were modeled with a duration of 1 s. Question trials were modeled with a duration of 3 s and video epochs were modeled with the duration of the respective video clip. The model equation consisted of the observed data, the design matrix, and the error-term.

For each participant, contrast images were generated, which consisted of beta-value estimates of the raw-score differences between experimental conditions. Subsequently, the individual contrast images were entered into a second-level random effects analysis. Here, one-sample *t*-tests across the contrast images of the 15 participants were performed to test the observed differences for significant deviations from zero. The *t*-values were transformed afterwards into z-scores.

We corrected for multiple comparisons by applying a two-step correction approach. An initial z-threshold of 2.33 (*p* < 0.01, one-tailed) was defined in the first step. All voxels showing a positive activation above this threshold entered the second step of the correction. Here, a Monte Carlo simulation was used to define thresholds for cluster-size and cluster-value at a significance level of *p* < 0.05 (one-tailed). The combination of cluster size and cluster value decreases the risk of neglecting true activations in small structures. Thus, all reported activations were significant at *p* < 0.05, corrected for multiple comparisons at the cluster level.

#### ROI analysis

To test for activations in the anterior hippocampus, we performed an additional region of interest (ROI) analysis for conditional entropy. The ROI in the left anterior hippocampus was defined by averaging coordinates of peak activation reported in previous studies on predictability of sequences of visual stimuli (Strange et al., 2005; Harrison et al., 2006; Bornstein and Daw, 2012); coordinates for the ROI in the right anterior hippocampus were derived from the study by Strange and colleagues. Center of the ROI in the left anterior hippocampus was at *x* = −25, *y* = −16, *z* = −18, center of the ROI in the right anterior hippocampus was at *x* = 31, *y* = −17, *z* = −19. Both ROIs had a sphere of six adjacent voxels. One-sample *t*-tests were calculated over beta-values per participant and ROI to test for significant deviations from zero.

Additionally, we tested *post-hoc* for correlation between beta-values derived from the parametric contrast of conditional entropy and the degree of familiarity with the statistical structure as assessed by the two post-tests. We quantified the degree of familiarity with the statistical structure separately for both post-tests as difference between the maximal probability judgment (100 for the computer and 8 for the paper–pencil post-test) and the average absolute deviation of the probability judgments ($\hat{p}$) from the implemented probabilities (*p*) (Equation 4).

$$Familiarity=p_{max}-\frac{1}{n}\sum_{n}\left|{\hat{p}}_{n}-p_{n}\right|$$

Equation 4. Calculation of the degree of familiarity with the statistical structure. Parameter $\hat{p}$ describes participants' probability judgments, parameter *p* the implemented probabilities of the different transitions (*n*) and *p_max_* the maximal judgment in the respective test (with a value of 100 for the computer and 8 for the paper–pencil post-test).

## Results

### Behavioral results

During the fMRI, participants answered on average 34.47 out of 36 questions correctly (*SD* = 1.77). One participant answered only 30 questions correctly (*z*-value < −2) and was excluded from all further analyses.

In order to assess whether participants learned the statistical structure of the actions, two post-tests were conducted.

Regarding the paper–pencil post-test, two participants had to be excluded from analyses, as they were erroneously presented only five instead of six objects. For the remaining participants, the RM-ANOVA on the estimated probabilities was significant [*F*_(1.69, 20.26)_= 19.52, *p* < 0.001, η^2^_*p*_ = 0.62]. Planned comparisons between the different levels yielded significant differences between the levels of 1.0 and 0.75 [*t*_(12)_ = 2.50, *p* = 0.014, one-tailed] and the levels of 0.50 and 0.25 [*t*_(12)_ = 6.56, *p* < 0.001, one-tailed; see Table 1 for means and standard deviations].

**Table 1:** **Descriptive results of the two post-tests, separately for the five levels of implemented probabilities**.

**Implemented probability**	**Distributed crosses in paper–pencil post-test: Mean (*SD*)**	**Estimated percentages in computer post-test: Mean (*SD*)**
0	0.95 (0.22)	22.03 (7.76)
0.25	0.86 (0.46)	22.77 (9.35)
0.50	2.79 (0.70)	44.12 (11.10)
0.75	2.65 (1.20)	44.17 (17.81)
1.0	4.54 (2.33)	59.80 (23.74)

**Figure 3:**
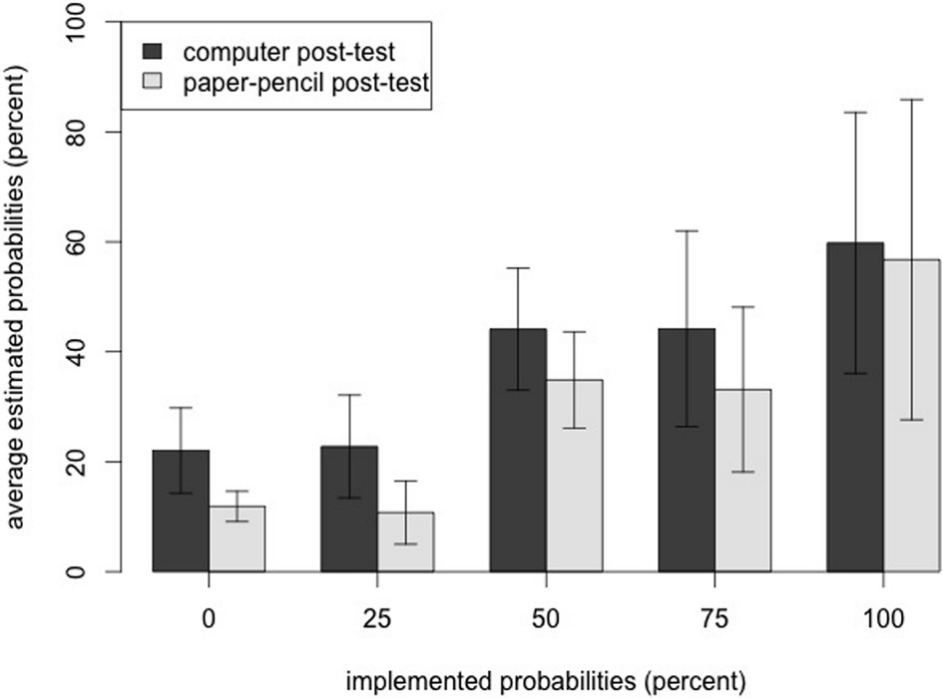
**Results of the two post-tests**. As one cross in the paper–pencil post-test corresponded to 12.5% in the computer post-test, results of the paper–pencil post-test were multiplied with the factor 12.5, to make participants' probability judgments in the two post-tests more comparable. Error bars display ± 1 *SD*.

The RM-ANOVA on the estimated probabilities of the computer post-test was also significant [*F*_(2.43, 34.05)_= 26.90, *p* < 0.001, η^2^_*p*_ = 0.66]. Two out of four planned comparisons between the different levels of probability reached significance: 1.0 vs. 0.75 [*t*_(14)_ = 3.36, *p* = 0.003, one-tailed] and 0.50 vs. 0.25 [*t*_(14)_ = 6.1, *p* < 0.001, one-tailed; see Table 1 for means and standard deviations of the five levels, Figure 3 for a graphical display].

Together, both post-tests consistently showed that participants rated transitions with higher probabilities as more likely, while they were not able to exactly distinguish between each probability level.

### Imaging results

#### Parametric effects of conditional surprisal

**Figure 4:**
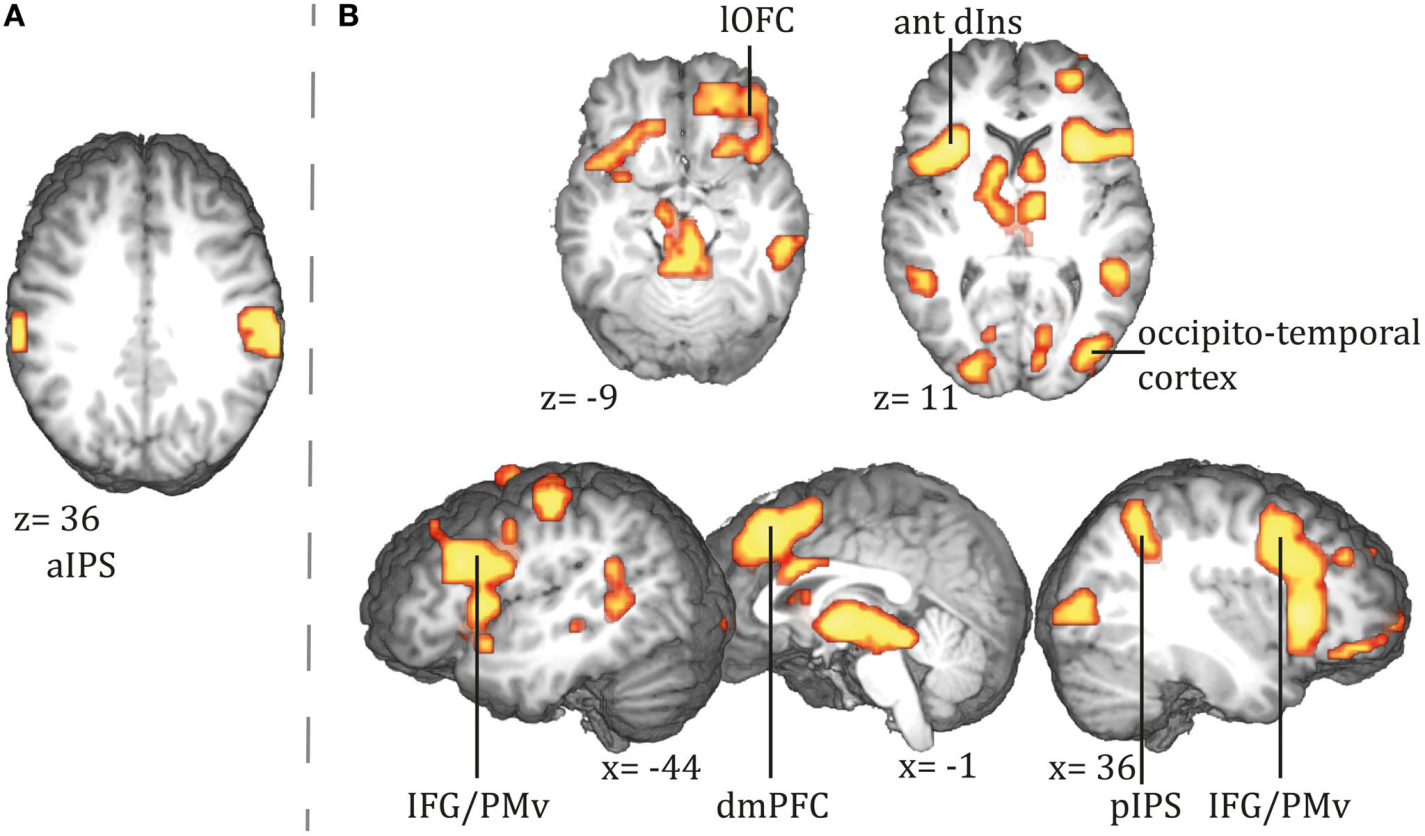
**Areas showing a positive correlation with (A) conditional surprisal and (B) conditional entropy**. ant dIns, anterior dorsal insula; dmPFC, dorsomedial prefrontal cortex; IFG, inferior frontal gyrus; lOFC, lateral orbitofrontal cortex; pIPS, posterior intraparietal sulcus; PMv, ventral premotor cortex; aIPS, anterior parietal sulcus.

Assessing parametric effects of conditional surprisal revealed a positive correlation in the bilateral anterior intraparietal sulcus (see Figure 4A and Figure S3 for additional sagittal views; a comprehensive list of activations and Talairach coordinates are provided in Table 2, see Table S5 for MNI coordinates).

**Table 2:** **Talairach coordinates and maximal z-scores of significantly activated voxels for the parametric contrasts of conditional surprisal and conditional entropy (combined GLM approach)**.

**Localization**	**Talairach coordinates**			***z*-values, local maxima**
	***x***	***y***	***z***	
**CONDITIONAL SURPRISAL**				
Anterior intraparietal sulcus/postcentral gyrus	55	−24	39	4.28
	−59	−27	36	3.08
**CONDITIONAL ENTROPY**				
Dorsomedial prefrontal cortex	1	24	45	4.43
Postcentral gyrus	−47	−18	54	3.96
Anterior dorsal insula	31	18	6	5.15
	−29	21	9	5.28
Posterior intraparietal sulcus	40	−48	33	3.51
Inferior frontal sulcus/ventral premotor cortex	−44	21	27	4.24
Anterior cingulate cortex	−5	15	24	3.65
Middle frontal gyrus	22	48	21	3.54
Posterior superior temporal sulcus	40	−48	33	3.51
	−47	−45	27	2.86
Inferior colliculi	1	−39	−6	4.21
Lateral temporo-occipital cortex	34	−81	9	3.29
Dorsal medial thalamus	−11	6	6	3.14
Cuneus	−23	−90	3	3.67
Medial orbitofrontal cortex	16	39	−9	3.55
Lateral orbitofrontal cortex	31	36	−12	3.43
	−29	3	−12	3.74

#### Parametric effects of conditional entropy

We found a positive correlation of conditional entropy with BOLD response in the right lateral and medial orbitofrontal cortex (lOFC and mOFC, hereafter), dmPFC, bilateral inferior frontal gyrus (IFG), bilateral anterior dorsal insulae, and right posterior intraparietal sulcus (pIPS) (see Figure 4B); a comprehensive list of activations and Talairach coordinates are provided in Table 2, see Table S5 for MNI coordinates.

#### ROI analysis

No significant hippocampal activation was revealed by the ROI analysis (all *p* > 0.4; see Table 3 for descriptive statistics of beta weights). The *post-hoc* correlation analysis revealed a significant positive correlation between familiarity with the statistical structure, measured as average deviation from the implemented probabilities (see Equation 4), in both ROIs when familiarity was assessed with the computer post-test (all *p* < 0.05), but not when it was assessed with the paper–pencil post-test (all *p* > 0.33, see Table 4). This correlation indicates that activation in the hippocampal ROIs was the stronger positively correlated with conditional entropy, the better participants had learnt the statistical structure of the action sequences.

**Table 3:** **Results of the ROI analyses in the left and right anterior hippocampus to test for effects of conditional entropy**.

**Region**	***t***	***df***	***p* (two-tailed)**	**Mean**	***SD***
Right hippocampus	−0.23	14	0.82	−0.05	0.85
Left hippocampus	−0.86	14	0.40	−0.15	0.66

**Table 4:** **Results of the correlational analysis between participants' knowledge of the statistical structure and beta-values derived from hippocampal ROIs**.

**Region**	**Computer post-test**				**Paper–pencil post-test**			
	***t***	***df***	***p* (two-tailed)**	***r***	***t***	***df***	***p* (two-tailed)**	***r***
Right hippocampus	3.80	13	0.002	0.73	−0.30	11	0.772	−0.09
Left hippocampus	2.22	13	0.045	0.52	−1.02	11	0.331	−0.29

## Discussion

From a stochastic point of view, the course of an action can be conceived of as a run through a decision tree: one step follows another with a certain probability while more or less alternative action steps are possible. In the present fMRI study we assumed that an action's statistical structure is reflected in the brain activity of the action observer. In particular, we aimed at deciphering two distinct aspects of the statistical structure that may influence processing of action steps. First, the load of this processing varies as a function of the action step's absolute probability at the point of the sequence, and hence unexpectedness or conditional surprisal. Second, the observed action step is more or less predictable, depending on the degree of branching of the decision tree at the considered action boundary and the probability weights of these different branches. This latter characteristic can be quantified as conditional entropy, which is higher, the less predictable an upcoming action step is.

We employed an ideal Bayesian observer model and analyzed the BOLD response for (1) the conditional surprisal and (2) the conditional entropy at beginnings of action steps. We found activation in the aIPS to positively correlate with an action step's conditional surprisal. For conditional entropy, we expected a positive correlation with activity of the AG, the hippocampal formation, and the dmPFC. We found activation in the right dmPFC and in the pIPS, close to the AG. No effect in the hippocampal formation was found. Instead, activity also increased with conditional entropy in the right lOFC and the bilateral anterior dorsal insulae. Findings will be discussed in detail below.

### Behavioral findings: participants' awareness of probabilistic action structure

A post-fMRI survey revealed that participants had little awareness of the probabilistic structure of the actions. However, they were able to report those pairwise associations between the action steps with highest transition probabilities (board–cube, short screw–long screw, washer–screw nut). No participant reported having noticed the different probabilities or degrees of predictability. Still, the two post-tests showed that participants implicitly learned the transition probabilities, as more likely transitions were judged to occur with a higher probability than less likely transitions. These judgments delivered in a computer test were moreover confirmed in a subsequent paper–pencil test, in which more likely transitions were selected more often than less likely transitions. Behavioral data indicate that the probabilistic structure of observed action is acquired and retrieved during action observation, even if not explicitly attended or consciously perceived.

The present findings add up to studies on statistical learning in actions (Avrahami and Kareev, 1994; Baldwin et al., 2008). Previous findings suggested that human observers can distinguish between random and deterministic transitions between distinct video clips showing object manipulations or movie excerpts. Our results indicate that human observers are also sensitive to statistical structure within continuous action sequences; furthermore, they are able to distinguish between different degrees of transition probabilities between action steps. This means that human observers can detect meaningful segments within uniform streams of actions based on statistical information. Critically, we did not distinguish between transitions between objects and transitions between object manipulations. Further studies should test for potential differences in effects of transition probabilities.

### Conditional surprisal: probability-dependent engagement of the anterior intraparietal sulcus

Expectations can serve as a filter for sensory input, inasmuch as everything that accords to the expectations is largely uninformative. By filtering on an early stimulus processing level, unexpected and hence informative sensory signals get more accentuated (Wolpert and Flanagan, 2001; Friston and Kiebel, 2009; Summerfield and Egner, 2009). This results in greater neural activity for unexpected events compared to expected events in stimulus- and task-relevant brain areas. As observing actions is known to engage the lateral network of premotor cortex, parietal sites, and posterior temporal cortex (Jeannerod, 2001; Schubotz and von Cramon, 2004; Caspers et al., 2010), we expected effects of conditional surprisal of an action step to be found here. Importantly, all action steps in the present study had an equal base rate, so that effects were not due to unexpectedness of the action step *per se*. Rather, we tested if expectations can be built based on transition probabilities between action steps. If so, action steps with a higher conditional surprisal should accordingly elicit a higher BOLD response in the action-observation network, as they were comparatively unexpected at that very point in time, and the conveyed information would not have been selectively filtered in advance (Schiffer et al., 2013).

We found enhanced BOLD response for unexpected action steps in the aIPS. The anterior portion of the IPS has been supposed to be the homolog to area AIP in macaques, which is sensitive to size, shape, and orientation of objects (Grefkes and Fink, 2005). In humans, it is proposed to deal with processing of tactile and visual object properties (Grefkes and Fink, 2005) and has been related to the online control of grasping and coding for goals in actions (Hamilton and Grafton, 2006; Tunik et al., 2007). Furthermore, aIPS together with temporo-occipital sites and the premotor cortex forms a network which is most commonly activated during action observation (Caspers et al., 2010). In accordance with current accounts of predictive coding during action observation, processing in this network is hierarchical (Kilner et al., 2007), meaning that information about object properties is fed forward from temporo-occipital sites to aIPS and from there to the premotor cortex. While activation in PMv and temporo-occipital cortex was modulated by conditional entropy of action steps, activation in aIPS covaried with unexpectedness of action steps. We suggest that increased activation in the aIPS for unexpected action steps reflect a revision of the previously built sensorimotor forward model of the expected manipulation of the object.

Given that we found the expected covariation with conditional surprisal of an observed object manipulation in the aIPS, it remains unclear why there was no such effect in earlier visual areas. It is possible that the statistical structure was not assigned to the relation between successively manipulated objects, but rather between successively performed action steps, i.e., a compound of object and its manipulation. A revision of the built forward model should draw to a larger extend on aIPS than temporo-occipital areas. In line with this, Schubotz and von Cramon (2008) found activation of aIPS in a switching paradigm particularly then when both the goal of an action as well as the involved object remained the same, while it did not reach significance anymore when one of the two changed. The aIPS was the only component of the action observation network that showed this activation pattern, while PMv was also significantly active when the goal of an action changed and temporo-occipital sites when changes of objects occurred. Thus, aIPS seems to be specifically sensitive to the compound of objects and their associated manipulations. It should be noticed that participants were instructed to answer questions regarding the used objects, rather than the different manipulations. Though we cannot conclude for sure that the observed effects relate to action observation rather than object observation, activation in the aIPS suggests that attention was nevertheless directed at the action step as a whole, instead of focusing on the objects alone.

Alternatively, the revealed effect of conditional surprisal could also be explained using the concept of expectation attenuation. Expectation attenuation describes a reduced neural response to an expected compared to an unexpected stimulus (Den Ouden et al., 2010; Todorovic and de Lange, 2012). It is comparable to effects of repetition suppression, but in contrast to this, it does not rely on direct repetitions of stimuli, but expectations based on memory. Our findings coincide also with results reported by Strange et al. (2005). The authors presented sequences of visual stimuli while participants had to respond to each stimulus by a corresponding button press. They found that activation in posterior fusiform gyrus and aIPS increased with increasing stimulus-induced surprisal. In contrast to the present study, the degree of surprisal in the study by Strange and coworkers depended on the overall probability of one stimulus to occur, i.e., its base-rate, and not on the current probability of a stimulus given the preceding one. Notably, since stimulus identity and required responses were not separated in the study by Strange and colleagues, their findings cannot clearly distinguish if the revealed neural response to stimuli with a higher surprisal reflects revision of prepared responses or revision of anticipated stimuli. In the present study, participants did neither have to respond to single action steps nor where they instructed to attend to the structure of the sequences. Higher activation of the aIPS for action steps with higher surprisal thus suggests that it also engages in predictive processes during passive observation of actions. Together, present findings and findings by Strange and colleagues indicate that the aIPS might be sensitive to the degree of surprisal in both dynamic as well as static visual sequences. Future work is needed to clarify if it is particularly sensitive to base-rate dependent surprisal of events, conditional surprisal, or both and how the effects are modulated by participants' task.

A possible alternative interpretation of activation patterns revealed by conditional surprisal in the present study would hold that it merely reflect visual processes. Possibly, participants used their knowledge of the most probable next object to focus their attention on it before action onset. In cases of surprising action steps, attention then would have to be withdrawn from the previously attended object and reoriented to the actually grasped one. Attentional reorienting, as for example necessary in the Posner paradigm, has been shown to correlate with activation in the superior temporal lobe and the inferior parietal cortex (Vossel et al., 2006). Accordingly, the correlation between conditional surprisal and aIPS activation may reflect attentional reorienting rather than revisal of anticipated action steps. However, this interpretation is unlikely for two reasons. Firstly, during all movie scenes presented in the current study, numerous exemplars of the different objects were concurrently visible, so that focusing attention on only one exemplar seems a highly implausible strategy. Secondly, the aIPS effect was specific for surprisal and did not overlap with any activation correlated with conditional entropy. If it would reflect necessity of attentional withdrawal, aIPS should be also modulated by the degree of conditional entropy, since conditional entropy describes how likely a shift of attention will be.

To sum up, finding activity in the aIPS to increase with a probabilistic mismatch between expected and observed action is in line with previous research showing it together with posterior temporal and premotor cortex to be activated when observed actions violate the observer's expectations (Schubotz and von Cramon, 2008; Schiffer et al., 2013) as well as when abstract visual stimuli elicit surprise (Strange et al., 2005; Bubic et al., 2011). The present results extend our knowledge on mechanisms underlying action observation by showing that expectations regarding upcoming action steps are constantly built and adapted during sequences of actions, even if not relevant for a task, and that the sensorimotor network is moreover sensitive to the strength of an observer's current expectations.

### Conditional entropy: predictability of action steps

Predictability can be viewed as the backdrop on which an occurring action step is processed. Thus, two action steps can be equally expected (in terms of their absolute probability), but for one action step, only one alternative action step exists (so that overall predictability is high) whereas for the other action step, several alternative action steps are concurrently possible (so that overall predictability is low). Accordingly, predictability is influenced by the number and the probabilities of all alternatives at a given point in an action.

We used conditional entropy to quantify action predictability, combining the number of possible action steps and their respective probabilities. Conditional entropy is the inverse of predictability, i.e., it is higher, the lower the predictability is. Several of our hypotheses, but not all, were confirmed by the data.

As expected, we found that high conditional entropy (and hence low predictability) of the next action step correlated positively with the BOLD response in the dmPFC, as well as anterior dorsal insulae, and lateral prefrontal cortex. Activation of the dmPFC, together with the anterior dorsal insula, is often found to increase during decision-making under uncertainty (Huettel et al., 2005; Volz et al., 2005; Preuschoff, 2008), also when uncertainty is unrelated to a possible outcome but affects a perceptual decision (Grinband et al., 2006; Summerfield et al., 2011). Uncertainty can be due to two factors; one is a lack of knowledge of the rules which describe the relation between events, also referred to as internally attributed uncertainty, the other is due to non-deterministic, i.e., probabilistic, relations between events, so that even when the rules which describe their relation are perfectly known, a perfect prediction of the upcoming event is not feasible. The latter type is also referred to as externally attributed uncertainty (Volz et al., 2005). Externally attributed uncertainty is induced by conditional entropy, as both rise as the number of possible events as well as the balance of their (reward) probability increases (Hirsh et al., 2012). It has been proposed that anterior dorsal insula sub-serves a translation from unspecific drive states to concrete action plans when uncertainty is high (Wager and Barrett, 2004). For an actor, uncertain situations call for preparation of alternative actions and a flexible shifting between action plans. In the present study, we suggest that a similar coping strategy may apply to action observers: if conditional entropy of an upcoming action step was high, this led to recollection of the known alternative action steps and the readiness to flexibly shift between them in the course of action analysis, reflected in an increased BOLD response in anterior insula. Please note that since we only manipulated the degree of conditional entropy of the statistical structure underlying the action sequences and did not assess participants experienced uncertainty or asked them to engage in predictions, it remains speculative whether insular activation is an indicator of participants' feeling of uncertainty. We suggest that physiological processes associated with conscious coping with uncertainty (as, for example, during decision making) and those triggered by observing action steps with high conditional probability might partially overlap.

Notably, in contrast to our findings, Bornstein and Daw (2012) found a linear negative, not positive, relation between conditional entropy and activation in the anterior insula as well as the prefrontal cortex. Furthermore, Tobia et al. (2012) reported that the response profile of the insula to entropy could be best explained by a step-down function. That is, the authors found that insular activation was higher when entropy levels were within the lower 25% of the distribution of employed sequential entropy, and lower for medium to high entropy levels. No linear relation between entropy and insular activation was found.

The obvious discrepancy between these two studies reporting a negative relation between insular activity and stimulus entropy and our findings of a positive correlation calls for an explanation. An obvious difference can be identified in the learning stages at which participants were tested in the studies of Bornstein and Tobia, and ours: implicit knowledge of the statistical structure and hence the conditional entropy assigned to upcoming action steps was already established in the present study when participants entered the fMRI session and was kept stable throughout the whole sessions. Though we cannot exclude that participants continued learning about the statistical structure, the situation did not call for an acquirement of new knowledge about the underlying structure, but rather for further adjustments of the already existing knowledge. In contrast, the statistical structures had to be learnt online in the studies by Bornstein and Tobia and also changed during the experiment. Hence, whereas upcoming action steps in the present study were unpredictable solely because of the underlying statistical structure of the action sequences, upcoming stimuli in the two other studies were unpredictable because of two factors, the probabilistic nature of the underlying structure as well as lacking (implicit) knowledge about the nature of the structure itself. Further studies have to evaluate if this psychological difference caused the divergent response profiles in anterior dorsal insula.

In sum, our findings corroborate the role of dmPFC and anterior dorsal insula in situations of low predictability. Crucially, participants in the present study were not explicitly asked to engage in predictions nor was the statistical structure of the observed action relevant to the task. Modulations in dmPFC and anterior dorsal insula activity therefore show that prediction is an automatically triggered process during action observation. Moreover, statistically induced fluctuations of predictability do not have to become conscious to participants to modulate activation in dmPFC and anterior dorsal insula.

Based on previous findings (Strange et al., 2005; Harrison et al., 2006; Bornstein and Daw, 2012; Schiffer et al., 2012), we expected to find a positive correlation between the BOLD signal in the hippocampal formation and the conditional entropy assigned to the upcoming action step. Data did not support this hypothesis. However, we found *post-hoc* a correlation between the hippocampal beta-values and participants' familiarity with the statistical structure: the better participants had learnt the statistical structure, the stronger was the hippocampus positively correlated with the conditional entropy (i.e., the higher was the extracted beta-value). It has been proposed that correlation between hippocampal activation and predictability reflects retrieval of mental representations of possible events (Bornstein and Daw, 2012; Schiffer et al., 2012), such that hippocampal activity increases with the number (and hence unpredictability) of possible events. The revealed finding here suggests that this correlation depended on the degree of implicit knowledge participants had acquired. However, given the correlational nature, the data can also be interpreted differently. Possibly, the correlation between conditional entropy and hippocampal activation did not result from participants' higher familiarity with the statistical structure, but was a prerequisite for it.

Note that the considered correlation was only found for the computer post-test, but not for the paper–pencil post-test on statistical action knowledge. We suggest that the two post-tests engaged different processes. The computer post-test was closer to the experimental requirements during the training and the fMRI session, since participants were presented with a short video clip showing the succession of two action steps. Furthermore, probability judgments were assigned to the just presented transition and participants were not required to take the alternative transitions into account as in the paper–pencil post-test. Thus, participants may have reflected their judgments more in the paper–pencil post-test, making it a more explicit knowledge test, relying also on different memory systems.

A positive yet un-hypothesized correlation between activation of the right lOFC and conditional entropy was revealed. Increased activation in lOFC has previously been reported for inference of action goals based on manipulation information (Schubotz and von Cramon, 2008). The authors suggested that activation of the lOFC reflects increased demands on evaluating which of the expected action goals fits best with the observed manipulation. In close keeping with this interpretation, we assume that lOFC weighs information on currently possible action steps and their respective probabilities to lateral and medial PFC. According to Wallis (2007), dlPFC and dmPFC use this information to generate cost-benefit balanced behavioral plans. With the proceeding of the action step, further sources of information, as, e.g., motion signals, including trajectories, hand postures, or grip type become available. Due to its connections to sensory areas, the OFC can integrate this information and provide this to dlPFC, further biasing the prediction of the action step (cf. Wallis, 2007). Accordingly, studies on decision-making report the lOFC for finding contingencies between stimulus-outcome associations (Rushworth et al., 2011) as well as for facing ambiguity, i.e., uncertainty due to missing information (Hsu et al., 2005). In these situations, further information, provided for instance by the reward history or somatic markers, has to be integrated to come to a decision (Bechara et al., 2000; Hsu et al., 2005; Mushtaq et al., 2011). Possibly, in the present study information on interoceptive states is provided by anterior dorsal insula (Craig, 2009).

Furthermore, we found activation in the pIPS to increase with conditional entropy. This activation points to altered attentional processes under low predictability. The pIPS belongs to the ventral frontoparietal network, as described by Corbetta and Shulman (2002). The authors proposed that the ventral frontoparietal network is particularly engaged in processing of previously unattended stimuli and hence reflects an orienting response to unexpected stimuli. Interestingly, we did not find activation in the pIPS modulated by conditional surprisal of an action step, but only by its conditional entropy. If conditional entropy is high, the likelihood of a necessary reorientation rises. We therefore speculate that activation of the pIPS in advance to necessary reorienting reflects a preparatory activation, dealing with the required flexibility of attention focus under high conditional entropy. This interpretation is in line with findings by Schubotz et al. (2012). In their study, activation of the posterior parietal cortex (more precisely, the posterior AG) was revealed when detection of action boundaries was contrasted with the detection of boundaries in intransitive (tai chi) movements. The authors suggest that at action boundaries, an exploration of potentially upcoming relevant aspects of the scene takes place and a shifting of attention to this spots is prepared, which is reflected in increased activation of posterior AG. We thus suggest that during action observation, participants' brains exploit scenes in anticipation of an upcoming reorientation of attention, resulting in a rise of activation in posterior parietal cortex.

To sum up, we found that conditional entropy of observed actions drew on areas known to be engaged during decision making under uncertainty, namely the dmPFC, anterior dorsal insula, and lOFC, as well as on the pIPS, an area that has been associated with shifts of attention. We suggest that pIPS reflects the preparation of potential shifts of attention when the further course of the action is rather unpredictable. Possibly, dmPFC, anterior dorsal insula, and lOFC show integration of additional information in order to enhance action prediction.

### Final remarks

The present fMRI study focused on the exploitation of the statistical structure in observed actions. We found that two characteristics can be distinguished with regard to their neural correlates. On the one hand, low predictability of action steps calls for a top-down modulation of attentional focus and stimulus processing, reflected in higher activation in a fronto-parietal network. On the other hand, low probability of an action step shows in a stronger accentuation of bottom-up signals provided by the stimulus, indicated by higher activation in parietal sites.

## Conflict of interest statement

The authors declare that the research was conducted in the absence of any commercial or financial relationships that could be construed as a potential conflict of interest.
