# Supplementary material for: Dissociating dynamic probability and predictability in observed actions—an fMRI study
Source: Front Hum Neurosci. 2014 May 7;8:273. doi: 10.3389/fnhum.2014.00273 (PMC4019881; doi:10.3389/fnhum.2014.00273)
Supplement: Supplementary file 1 [file DataSheet1.PDF]

Supplementary Material

Dissociating dynamic probability and predictability in observed actions – an fMRI study

Christiane Ahlheim 1[1,2] , Waltraud Stadler 2[3,4], Ricarda I. Schubotz3[1,2]

[1]Institute of Psychology, University of Münster, Münster, Germany,
[2]Motor Cognition Group, Max Planck Institute for Neurological Research, Cologne, Germany
[3]Department of Sport and Health Science, Technische Universität München, Munich, Germany
[3]Department of Cognitive Neurology, Max Planck Institute for Human Cognitive and Brain Sciences, Leipzig, Germany

*** Correspondence:** Christiane Ahlheim, Institute of Psychology, University of Münster, Fliednerstr. 21, 48149 Münster
christiane.ahlheim@uni-muenster.de

1. Functional Localizer

In addition to the main experimental block showing the 74 action sequences, we ran four functional localizers adapted from Wurm and Schubotz (2012) after the main experiment so as to identify brain regions related to the processing of Baufix® objects, other tools, motion, and human body. However, since our hypotheses only addressed effects of grasping and manipulating unexpected Baufix® objects, but not other tools, motion, or body parts, only the Baufix® localizer will be reported in more detail.

To identify regions sensitive to processing of Baufix® objects, we employed pictures showing the six different Baufix® objects used in this study: cube, washer, screw-nut, board, long screw, and short screw. To create a control condition with physically identical attributes, but no information on object identity, pictures showing scrambled Baufix® objects (with a grain of 5x5 pixels) were used. These two different stimulus types were presented in separate blocks. Both the Baufix® block and the Scrambled-Baufix® block consisted of 24 pictures each, generated by six different pictures that were presented four times in randomized order. Each picture was presented for 300ms and with an inter-stimulus interval of 450ms, resulting in a total block length of (6 * 4 * 750ms =) 18 seconds. Each block was presented three times, and a fixation circle separated blocks for 8 sec. The order of the blocks was counterbalanced across participants. Participants were instructed to press a button whenever the same picture appeared twice in a row (n-1), which happened one to three times per block. The participants received visual feedback on hits, misses, and false alarms (+, -, respectively).

For the statistical analysis of the fMRI effects of the functional localizer, a separate design matrix was generated for each participant. Analyzed epochs comprised the duration of blocks (18s) and were convolved with a Gaussian function. In order to identify the lateral occipital complex (LOC), blocks of Baufix® objects were contrasted with blocks of Scrambled-Baufix® objects. Contrast images were generated for each participant that consisted of beta value estimates of the raw-score differences between blocks of pictures of Baufix® objects and blocks of scrambled Baufix® objects. Subsequently, the individual contrast images were entered into a second-level random effects analysis. Here, one-sample t-tests across the contrast images of the 15 participants were performed to test the observed differences for significant deviations from zero. The t-values were transformed afterwards into z-scores.

For a description of the employed correction for multiple comparisons, please refer to the main text.

1.1. Results

Contrasting blocks of pictures of Baufix® objects with blocks of pictures of scrambled Baufix® pictures revealed increased activation for Baufix® objects bilaterally in a lateral occipito-temporal region that we take to reflect the LOC (see Figure S1, a comprehensive list of activations and Talairach coordinates are provided in Table S1).

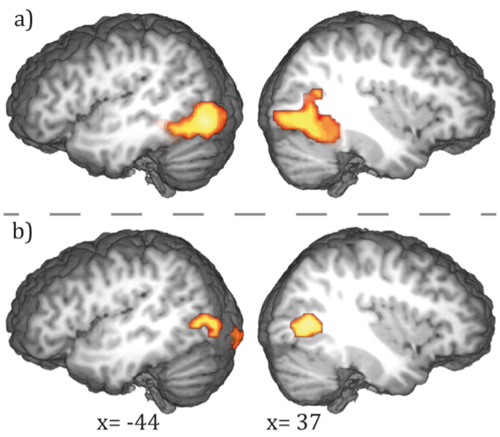

Figure S1: Lateral occipital cortex, a) as identified by the localizer, b) corresponding areas showing a positive correlation with conditional surprisal.

Table S1: Talairach coordinates and maximal z-scores of significantly activated voxels of the contrast Baufix® > Scrambled-Baufix®

Localization	Talairach coordinates			z-values,	mm^3
	x	y	z	local maxima	
Lateral occipito-temporal complex (LOC)	31	-84	0	4.21	8424
	-44	-72	-3	5.23	12609
Posterior cingulate cortex	-5	-54	30	4.24	9153

2. Separate GLM analysis for effects of conditional entropy and conditional surprisal

Modelling the correlated parametric regressors for conditional entropy and surprisal simultaneously in one GLM puts the risk that areas that are truly modulated by both of them do not show up as significantly activated because they cannot be uniquely ascribed to one of them. To identify false negatives in the reported z-maps (see main text), we additionally employed separate

analyses for both of them. Apart from including only one parametric regressor, the analyses were identical to the one reported in the main text.

The design matrices included five regressors: one for the main effect of action onsets with an amplitude of one, one for the parametric effect of conditional entropy or conditional surprisal, respectively, with an amplitude corresponding to the respective measure, and two each with an amplitude of one for question trials and video epochs. We added the duration of the action steps as regressor of no interest. Besides the video epochs and the question trials, all events were modeled with a duration of one second. Question trials were modeled with a duration of three seconds and video epochs were modeled with the duration of the respective video clip. We again applied a two-step correction for multiple comparisons, by applying first an initial z-threshold of 2.33 ($p<.01$, one-tailed) and then defining thresholds for cluster-size and cluster-value at a significance level of $p<.05$ (one-tailed), using a Monte-Carlo simulation.

We again tested for effects of conditional entropy in the hippocampal ROIs, as described in the main text.

2.1. Results

We found a positive correlation between conditional surprisal and activation in the bilateral occipito-temporal cortex. This activation overlapped to 65.9 % in the left and 53.3 % in the right hemisphere with activation revealed by the functional localizer of LOC (see Figure S1 and S2; a comprehensive list of activations and Talairach coordinates are provided in Table S2). In contrast to the combined GLM analysis, no significant activation in the parietal cortex was revealed when modeling conditional surprisal separately.

The activation pattern revealed by separately analyzing effects of conditional entropy largely matched the activation pattern revealed by the combined analysis (see Figure S2; a comprehensive list of activations and Talairach coordinates are provided in Table S2). However, we did not find a significant positive correlation between conditional entropy and activation in the posterior IPS.

Findings from our ROI analysis were also highly similar to the results revealed by the combined analysis (see Table S3 and S4).

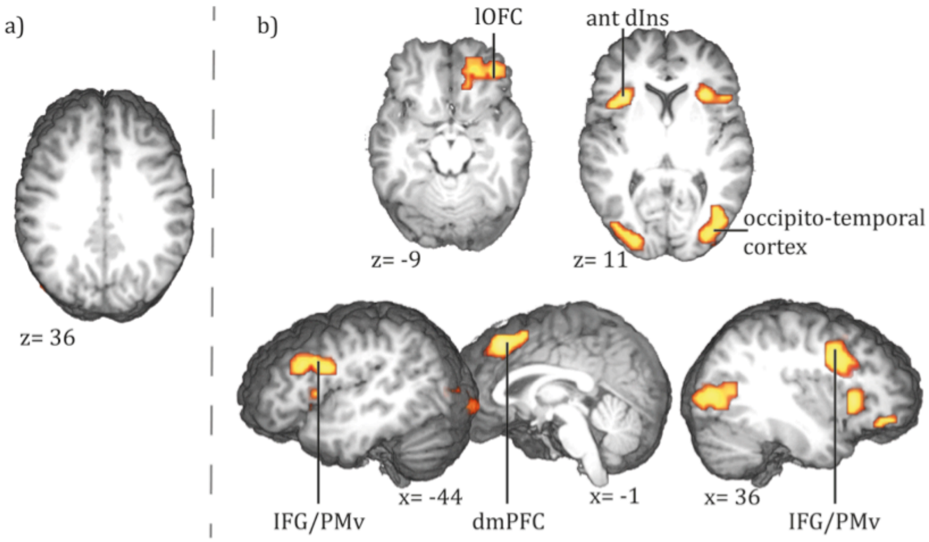

Figure S2: Areas showing a positive correlation with a) conditional surprisal and b) conditional entropy, when modeled separately. ant dIns: anterior dorsal insula; dmPFC: dorsomedial prefrontal cortex; IFG: inferior frontal gyrus; lOFC: lateral orbitofrontal cortex; PMv: ventral premotor cortex.

Table S2: Talairach coordinates and maximal z-scores of significantly activated voxels for the parametric contrasts of conditional surprisal and conditional entropy (separate GLM approach).

Localization	Talairach coordinates x	y	z	z-values, local maxima
Conditional surprisal				
Lateral occipito-temporal complex (LOC)	34	-63	9	3.99
	-26	-90	-3	3.27
Conditional entropy				
Dorsomedial prefrontal cortex	1	24	45	4.27
Inferior frontal sulcus/ ventral premotor cortex	34	3	33	3.39
	-41	6	27	3.29
Middle frontal gyrus	-44	21	27	3.34
Anterior dorsal insula	-29	21	9	4.51
	28	18	3	4.23
Lateral temporo-occipital cortex	37	-75	9	3.50
	-26	-87	3	3.83
Lateral orbitofronal cortex	31	39	-9	3.74
Medial orbitofrontal cortex	16	39	-9	3.35

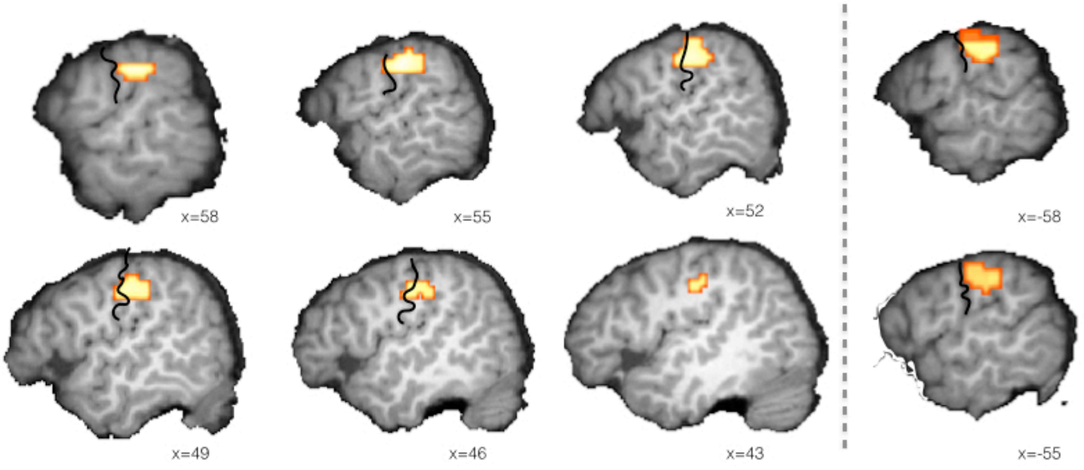

Figure S3: Sagittal views of the effects revealed by the parametric contrast of conditional surprisal (combined GLM approach). The black line highlights the postcentral sulcus.

Table S3. Results of the ROI analyses in the left and right anterior hippocampus to test for effects of conditional entropy (separate GLM approach).

Region	t	df	p (two-tailed)	Mean	SD
Right hippocampus	-0.40	14	.698	-0.07	0.66
Left hippocampus	-0.50	14	.624	-0.07	0.57

Table S4. Results of the correlational analysis between participants' knowledge of the statistical structure and beta-values derived from hippocampal ROIs (separate GLM aaproach).

	Computer post-test				Paper-pencil post-test			
Region	t	df	p (two-tailed)	r	t	df	p (two-tailed)	r
Right hippocampus	3.70	13	.003	.72	-0.40	11	.70	-.12
Left hippocampus	2.38	13	.033	.55	-1.20	11	.254	-.34

3. Discussion

The separate GLM approach on entropy and surprisal largely confirmed findings revealed by the combined GLM (cf. main text). However, activation in the parietal cortex which were revealed by the combined analysis for both entropy and surprisal did not reach significance when parameters where modeled separately. It therefore appears that the respective other parameter improved detection power in parietal cortex by explaining systematic variance, hence reducing the amount of unexplained variance, which functions as the error term in the GLM. This means that different aspects of variance in parietal cortex can be explained by conditional entropy and surprisal. Including both of them controls for this variance, which is due to conditional entropy and surprisal, so that overall the error term becomes smaller and detection power increases.

Contrary to the combined GLM, we found activity in the occipito-temporal region (roughly LOC, according to our functional localizer) to increase with conditional surprisal, but only when conditional surprisal was modeled separately. However, as the combined analysis shows, this area was even more ruled by conditional entropy (unpredictability) of action steps. While under high predictability (i.e. low conditional entropy), a strong sensorimotor forward model can be built which attenuates incoming sensory signals (cf. Friston and Kiebel, 2009; Schiffer et al., 2013), under low predictability a higher number of forward models have to be generated, resulting in a greater activation in LOC and less effective filtering. Because of this modulation of LOC activation due to conditional entropy, further effects due to the unexpectedness per se could be cancelled out.

Table S5: MNI transformed coordinates and maximal z-scores of significantly activated voxels for the parametric contrasts of conditional surprisal and conditional entropy (combined GLM approach).

Localization	MNI coordinates			z-values,
	x	y	z	local maxima
Conditional surprisal				
Anterior intraparietal sulcus	61.10	-19.81	40.04	4.28
	-62.09	-23.74	39.16	3.08
Conditional entropy				
Dorsomedial prefrontal cortex	2.65	31.66	42.66	4.43
Postcentral gyrus	-48.91	-12.26	57.97	3.96
Anterior dorsal insula	34.54	21.34	-0.56	5.15
	-30.25	24.62	3.58	5.28
Posterior intraparietal sulcus	44.91	-46.01	36.18	3.51
Inferior frontal sulcus/ventral	-46.21	26.43	23.84	4.24
Anterior cingulate cortex	-4.09	19.88	20.41	3.65
Middle frontal gyrus	24.90	54.77	13.10	3.54
Posterior superior temporal sulcus	44.91	-46.01	36.18	3.51
	-49.18	-43.78	30.84	2.86
Inferior colliculi	2.20	-40.64	-7.33	4.21
Lateral temporo-occipital cortex	38.23	-83.63	13.12	3.29
Dorsal medial thalamus	-10.79	8.42	1.49	3.14
Cuneus	-23.40	-94.04	8.48	3.67
Medial orbitofrontal cortex	18.04	42.06	-19.14	3.55
Lateral orbitofrontal cortex	34.22	38.62	-22.44	3.43
	-30.47	3.29	-17.84	3.74

Note. Transformation to MNI space was done using the icbm2tal transform (Lancaster et al., 2007).

References

Friston, K. J., and Kiebel, S. (2009). Predictive coding under the free-energy principle. *Philos. Trans. R. Soc. Lond. B. Biol. Sci.* 364, 1211–1121. doi:10.1098/rstb.2008.0300.

Lancaster, J. L., Tordesillas-Gutiérrez, D., Martinez, M., Salinas, F., Evans, A., Zilles, K., Mazziotta, J. C., and Fox, P. T. (2007). Bias between MNI and Talairach coordinates analyzed using the ICBM-152 brain template. *Human Brain Mapping* 28, 1194–205. doi:10.1002/hbm.20345.

Schiffer, A.-M., Ahlheim, C., Ulrichs, K., and Schubotz, R. I. (2013). Neural changes when actions change: adaptation of strong and weak expectations. *Human Brain Mapping* 34, 1713–1727. doi:10.1002/hbm.22023.
